# What do we actually know about exosomal microRNAs in kidney diseases?

**DOI:** 10.3389/fphys.2022.941143

**Published:** 2022-08-29

**Authors:** Qianyu Li, Zhiping Zhang, Min Yin, Cancan Cui, Yucheng Zhang, Yali Wang, Feng Liu

**Affiliations:** ^1^ Department of Nephrology, China–Japan Union Hospital of Jilin University, Changchun, China; ^2^ Clinical Laboratory, China–Japan Union Hospital of Jilin University, Changchun, China; ^3^ Scientific Research Center, China–Japan Union Hospital of Jilin University, Changchun, China; ^4^ Department of Blood Transfusion, China–Japan Union Hospital of Jilin University, Changchun, China

**Keywords:** exosome, microRNA, kidney disease, biomarkers, physiology

## Abstract

There are several types of kidney diseases with complex causes. If left untreated, these diseases irreversibly progress to end-stage renal disease. Thus, their early diagnosis and targeted treatment are important. Exosomes—extracellular vesicles released by a variety of cells—are ideal carriers for DNA, RNA, proteins, and other metabolites owing to their bilayer membranes. Studies have shown that almost all renal cells can secrete exosomes. While research on exosomal microRNAs in the context of renal diseases begun only recently, rapid progress has been achieved. This review summarizes the changes in exosomal microRNA expression in different kidney diseases. Thus, it highlights the diagnostic and prognostic value of these exosomal microRNAs. Further, this review analyzes their roles in the development of different kidney diseases, guiding research on molecular mechanisms and therapeutic strategies.

## Introduction

Kidney diseases are one of the most common diseases around the world, affecting approximately 850 million people worldwide (Jager et al., 2019). There are several types of kidney diseases, and many of these have complex causes and a long course. If timely treatment is not administered, all kidney diseases irreversibly progress to chronic kidney disease (CKD) or end-stage renal disease. CKD is expected to become the fifth leading cause of death by 2040 (Foreman et al., 2018), creating a huge medical and economic burden globally. Hence, the early diagnosis and targeted treatment of kidney diseases are very important.

In humans, <2% of genomic DNA encodes proteins. For decades, research on disease mechanisms has focused on protein-coding genes. With the development of sequencing technologies, researchers have gradually discovered that many non-coding RNAs (ncRNAs) play important roles in the physiological and pathological processes of diseases. Growing evidence shows that ncRNAs—including tRNAs, nsRNAs, microRNAs (miRNAs), long non-coding RNAs (lncRNAs), and circular RNAs (circRNAs) — can regulate gene expression at multiple levels by interacting with DNA, RNA, and proteins (Holoch and Moazed, 2015).

MicroRNAs are a class of evolutionarily conserved, single-stranded non-coding RNAs and are approximately 18–24 nucleotides in length. MicroRNAs bind to the 3′-untranslated region (3′-UTR) of specific target mRNA, blocking mRNA translation and/or promoting mRNA degradation, consequently regulating gene expression at the post-transcriptional level (Krol et al., 2010). A specific microRNA may bind to and regulate multiple target mRNAs. Moreover, the 3′-UTR of a given mRNA may contain binding sites for several microRNAs, thus adding multiple levels of regulation (Lian et al., 2017).

Exosomes are extracellular vesicles, 40–160 nm in diameter, and are released by a variety of cells. Because of their bilayer membranes, exosomes act as ideal carriers for DNA, RNA, proteins, and other metabolites (Lian et al., 2017). Exosomes were once considered molecular trash. However, they are now known to mediate intercellular communication. Exosomes selectively encapsulate specific molecules and deliver them to nearby or distant target cells. Thereby, they participate in multiple pathophysiological processes, including immune responses (Lian et al., 2017; Yan and Jiang, 2020), nervous system communication (Xu et al., 2017; Men et al., 2019), tumorigenesis and progression (Zhang and Yu, 2019), cardiovascular diseases (Sahoo et al., 2021), and inflammation (Zhang et al., 2019a; Jiang et al., 2019). The role of exosomes in these processes and the underlying mechanisms have garnered widespread attention among researchers. Although studies on exosomes and exosomal microRNAs in the context of kidney diseases started relatively recently, rapid progress has been achieved. Research has shown that almost all renal cells can secrete exosomes, suggesting that exosomes may play important roles in kidney diseases (Pisitkun et al., 2004). These hypotheses have been confirmed in subsequent studies.

This review summarizes data on exosomal microRNAs that are differentially expressed in various kidney diseases, highlighting their roles in disease development. This review could thus guide research into the pathogenesis, diagnosis, and treatment of kidney diseases.

## Exosomal microRNAs in CKD

CKD is a major threat to human health and is caused by the progression of multiple kidney diseases. CKD is defined as structural and functional renal impairment lasting more than 3 months and is associated with many symptoms, including proteinuria, abnormal urinary sediment, electrolyte imbalance, and other abnormalities. An unexplained decrease in the glomerular filtration rate (GFR; <60 ml/min/1.73 m^2^) for more than 3 months combined with abnormalities in pathological and/or structural findings and a history of renal transplantation can also be defined as CKD (Kidney and Kidney, 2013). The progression of CKD, especially to end-stage renal disease, greatly affects patient quality of life and increases the risk of cardiovascular diseases and mortality (Eckardt et al., 2013). Therefore, the prevention and treatment of CKD have attracted extensive attention from researchers. At the molecular level, it has been confirmed that exosomal microRNAs have a role in CKD development. Evidence shows that exosomal microRNAs could act as potential diagnostic and prognostic biomarkers, as well as therapeutic targets. Thus, these microRNAs have become a hot spot of CKD research.

### Exosomal microRNAs as biomarkers for the diagnosis and treatment of CKD

Exosomal microRNAs have been found to serve as biomarkers for CKD, playing important roles in the early diagnosis, clinical monitoring, and pathological analysis of CKD. Kumari et al. found that urinary exosomal miR-451 was significantly up-regulated in the early stages of CKD (serum creatinine <2.0 mg/dl) and was negatively correlated with eGFR. Thus, it could help in the early diagnosis and clinical monitoring of CKD (Kumari et al., 2020). Using the ncRNASeqScan algorithm, Rimpi et al. identified 30 differentially expressed urinary exosomal microRNAs that act as biomarkers for the early diagnosis of CKD. Among these exosomal microRNAs, miR-181a was found to be significantly down-regulated in all CKD groups, showing a diagnostic value at all stages of CKD (Khurana et al., 2017).

Cats are common companion animals and are also susceptible to kidney diseases. In one study, urinary exosomal miR-181a levels were also found to be significantly decreased in cats with kidney disease, and the urinary exosomal miR-181a/miR-let-7b and miR-181a/miR-10b ratios were significantly and positively correlated with blood urea nitrogen and serum creatinine levels. This study also found that urinary exosomal miR-let-7b, miR-22, and miR-26a levels were significantly down-regulated in cats in the early stages of kidney disease (Ichii et al., 2018).

Animal experiments show that the pathological characteristics of kidney damage vary in different companion animals. The glomerulus is more susceptible to damage in dogs, whereas the tubulointerstitium is more vulnerable in cats (Ichii et al., 2011). Conventional markers of kidney damage, such as creatinine and urine protein levels, cannot be used to identify the specific sites of renal damage. However, microRNAs have shown potential for such specific diagnosis in different animals. Ichii et al. found that urinary exosomal miR-26a and miR-10a/b levels were significantly up-regulated in dogs with renal disease. The down-regulation of miR-26a and miR-10a/b in the glomeruli and miR-10b in the tubulointerstitium were negatively associated with worsening renal function and histopathology. In contrast, the up-regulation of miR-21a in the tubulointerstitium was positively associated with worsening renal histopathology (Ichii et al., 2017). Similarly, miR-21 was found to be up-regulated in mice with podocyte injury and in urinary exosomes from CKD patients, and its levels were negatively correlated with eGFR (Lange et al., 2019). Thus, we speculate that the changes in microRNA levels could, to a certain extent, help in differentiating the pathological sites of kidney damage.

Renal fibrosis is a common outcome of CKD. Some urinary and circulating exosomal microRNAs are known to be altered during the process of renal fibrosis, indicating their value in the diagnosis of renal fibrosis. One study found that urinary exosomal miR-200b levels were positively correlated with eGFR in CKD patients and significantly decreased with the progression of renal fibrosis (Yu et al., 2018). Urinary exosomal miR-29c also appeared to be a diagnostic marker for renal fibrosis. Lv et al. were the first to report that urinary exosomal miR-29c levels are correlated with the tubulointerstitial fibrosis score (Lv et al., 2013). Consistent with this finding, another study demonstrated the correlation between miR-29c and cystatin C levels (marginal statistical significance) and the significant negative correlation of miR-29c levels with eGFR and the relative interstitial area. These findings indicated that miR-29c is more closely related to the later stages of renal fibrosis. Furthermore, exosomal miR-21 levels were found to be positively correlated with the tubulointerstitial damage index, suggesting that miR-21 is more closely associated with the early stages of renal fibrosis and could be used for monitoring renal tubulointerstitial injury (Lv et al., 2018a).

Changes in exosomal microRNA levels can also reflect therapeutic effects. Angiotensin receptor blockers (ARBs) and angiotensin-converting enzyme inhibitors (ACEIs) are currently the treatments of choice for preventing CKD progression (Snively and Gutierrez, 2004). DPP-4 inhibitors are a class of hypoglycemic agents that have shown therapeutic effects independent of hypoglycemia as well as hypotension in both diabetic and non-diabetic CKD (Alter et al., 2012). The level of urinary exosomal miR-29s is significantly decreased in CKD patients (Lv et al., 2013), and a similar decrease can be detected in rat models of CKD. In a mouse model of CKD, Telmisartan significantly restored urinary exosomal miR-29b and miR-29c levels and linagliptin restored miR-29c levels, suggesting that urinary exosomal miR-29b and miR-29c could be biomarkers for drug efficacy (Delic et al., 2020). Previous studies have demonstrated that the down-regulation of miR-29 enhances the TGF-β-induced expression of collagen type I and III in renal tubular cells (Liu et al., 2010), promoting podocyte apoptosis, proteinuria, and renal dysfunction (Lin et al., 2014). These studies also demonstrated the antifibrotic effects of ARBs as well as DPP-4 inhibitors (Delic et al., 2020). Consistent with these findings, herbal medicines for CKD were also found to change the level of exosomal microRNAs. Treatment with Jian-Pi-Yi-Shen formula (JPYSF), the main herbal formula for CKD, was found to significantly attenuate the down-regulation of four serum exosomal microRNAs associated with CKD (miR-192-5p, miR-194-5p, miR-802-5p, and miR-143-3p). Of these, miR-192-5p showed the greatest value as a diagnostic biomarker for CKD and as a biomarker for monitoring the therapeutic effect of JPYSF(30).

### Mechanistic role of exosomal microRNAs in renal fibrosis and CKD complications

Increased tubulointerstitial atrophy and fibrosis are the pathological features of CKD. Renal fibrosis is caused by the infiltration of inflammatory cells, activation and proliferation of fibroblasts, excessive production and deposition of extracellular matrix (ECM) components, and atrophy of peritubular capillaries, which result from various etiologies (Humphreys, 2018; Djudjaj and Boor, 2019). In unilateral ureteral obstructed (UUO) mouse kidneys and TGF-β-stimulated tubular cells, exosomal miR-21 levels are up-regulated. Increased miR-21 can accelerate the development of renal fibrosis by activating fibroblasts *via* the miR-21/PTEN/Akt pathway (Zhao et al., 2021). Similarly, exosomes from miR-374a-5p-modified mesenchymal stem cells (MSCs) can inhibit TGF-β1-induced fibrosis by regulating the MAPK6/MK5/YAP axis (Liang et al., 2022). Furthermore, Yang et al. exploited MSC-derived exosomes for delivering miR-186-5p agomirs into the obstructed kidneys of UUO mice. They found that the exosomal miR-186-5p could attenuate kidney injury and fibrosis by inhibiting ECM protein accumulation and the epithelial–mesenchymal transition (EMT) *via* Smad5 targeting. These findings could help reveal the role of MSC-derived exosomes in alleviating renal fibrosis in CKD(35). Previous studies have revealed that miR-150 can inhibit myocardial fibrosis (Shen et al., 2019); however, miR-150 seems to show the opposite effect on renal fibrosis. The production of exosomes that contain miR-150-5p was observed to increase under hypoxic conditions *in vivo*, promoting fibroblast activation by targeting suppressor of cytokine signaling 1 (SOCS1) *in vitro*, leading to renal fibrosis (Zhou et al., 2021). Guan et al. also found that exosomal miR-150, secreted from renal tubular epithelial cells (RTECs), can be endocytosed by fibroblasts. This miR-150 then promotes the activation and proliferation of fibroblasts and finally facilitates renal fibrosis (Guan et al., 2020).

**FIGURE 1:**
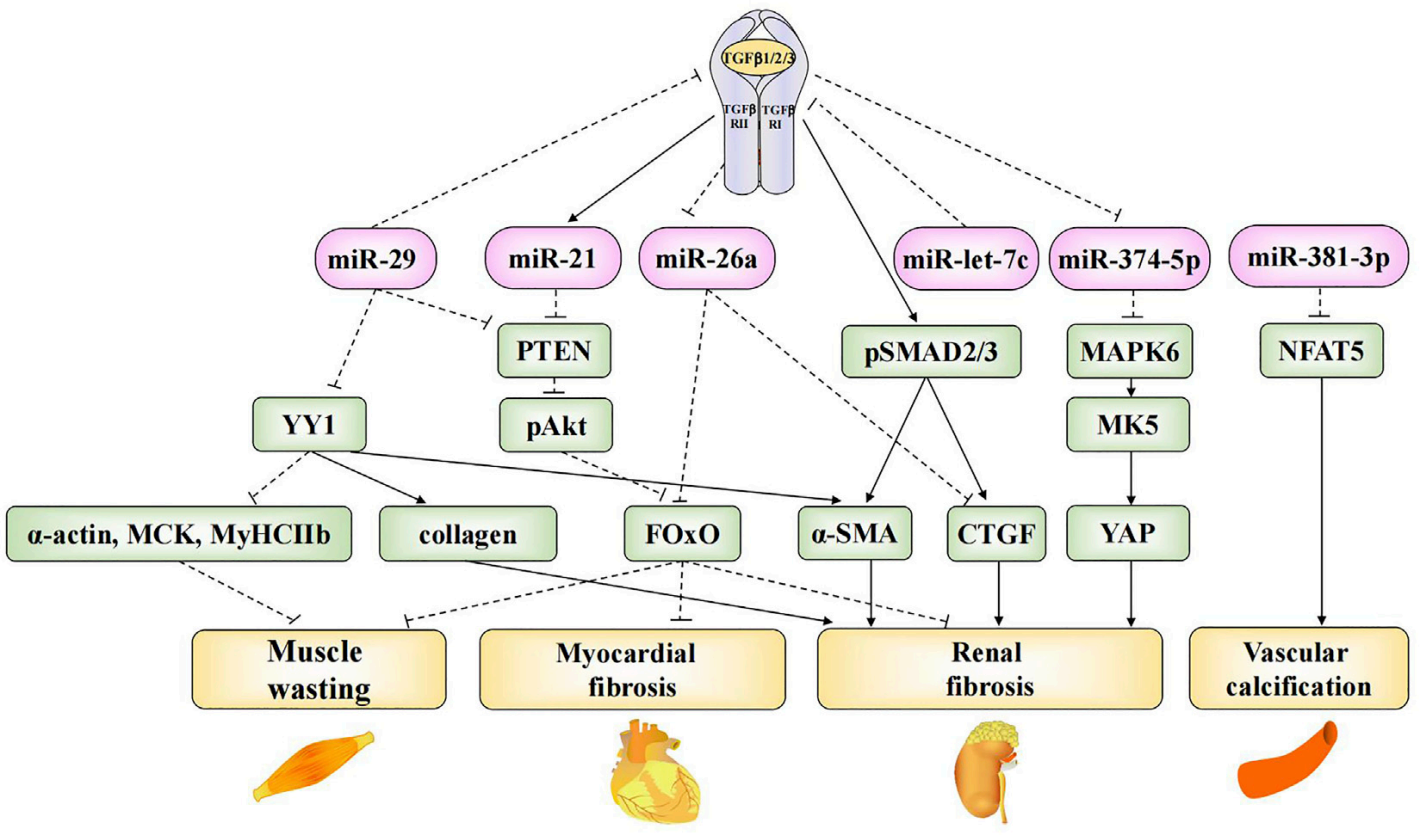
Mechanistic roles of exosomal microRNAs in TGF-β-mediated fibrosis and CKD complications. Exosomal miR-29, miR-21, miR-26a, miR-let-7c and miR-374-5p are involved in TGF-β-mediated fibrosis in CKD. Exosomal miR-29 and miR-26a are involved in the progress of muscle wasting. Exosomal miR-29, miR-21 and miR-26a are involved in the progress of myocardial fibrosis. Exosomal miR-381-3p is involved in the progress of vascular calcification.

Patients experience several systemic complications at the end-stage of CKD, including cardiovascular disease, neuromuscular disease, skeletal lesions, and endocrine disorders, which decrease quality of life and increase the risk of mortality. Vascular calcification is a common cardiovascular complication associated with CKD (Shroff et al., 2013). Bone marrow MSCs (BMSCs) are non-hematopoietic stem cells found in the bone marrow. Liu et al. found that BMSC-derived exosomal miR-381-3p could alleviate cellular apoptosis and vascular calcification by targeting NFAT5 (Liu et al., 2022). Myocardial fibrosis and muscle atrophy are the most extensively studied complications of CKD. Some exosomal microRNAs are involved in renal fibrosis as well as myocardial fibrosis and muscle atrophy. Previous studies have confirmed that the TGF-β signaling pathway promotes ECM synthesis and accumulation, leading to the hypertrophy and fibrosis of various cells. Thus, it plays a key role in the development of renal fibrosis (Trionfini et al., 2015). Many exosomal microRNAs were found to contribute to renal fibrosis through TGF-β-mediated signaling pathways; the roles of these microRNAs are summarized in Figure 1. Wang et al. found that exosomal miR-29 could directly inhibit the expression of TGF-β3 (Wang et al., 2019a), thereby ameliorating renal fibrosis. In another study, researchers injected a recombinant adeno-associated virus (AAV) containing a miR-29a overexpression construct into UUO mice. The overexpression of miR-29a reversed the up-regulation of the transcription factor Yin-Yang-1 (YY1), TGF-β, fibronectin, *α*-smooth muscle actin, collagen 1A1, and collagen 4A1 and ultimately inhibited renal fibrosis. Meanwhile, the overexpression of miR-29a suppressed muscle atrophy by inhibiting YY1, which directly targets the synthesis of various genes, including skeletal *α*-actin (α-actin) (Lee et al., 1992), muscle creatine kinase (MCK), and myosin heavy chain IIb (MyHCIIb) (Caretti et al., 2004). Furthermore, miR-29a overexpression was also found to inhibit the expression of PTEN, thus suppressing muscle atrophy and myocardial fibrosis *via* the PTEN/Akt/FOXO pathway (Wang et al., 2020).

The dephosphorylation-mediated activation of the transcription factor forkhead box O1 (FOxO1), which plays an important role in uremic muscle atrophy, induces muscle atrophy as well as the transcriptional up-regulation of E3 ubiquitin ligase, cardiac hypertrophy, and fibrosis (Wang et al., 2009; Xu et al., 2012). Exosomal miR-26a can prevent muscle atrophy and myocardial fibrosis by inhibiting FOxO1 (Wang et al., 2019b; Zhang et al., 2019b). Further, it can prevent renal fibrosis by directly inhibiting connective tissue growth factor (CTGF) (Zhang et al., 2019b), which contributes to the pro-fibrotic process through TGF-β (Koga et al., 2015). Finally, miR-let-7c is known to ameliorate renal fibrosis. Engineered MSC-derived exosomes that selectively deliver miR-let-7c to damaged kidney cells were found to significantly down-regulate collagen IV α1 and TGF-β type I receptor (TGF-β RⅠ) in UUO kidneys (Wang et al., 2016), thus preventing the progression of end-stage renal failure.

### Exosomal microRNAs in diabetic nephropathy

Diabetic nephropathy (DN) is a serious microvascular complication of diabetes and a major cause of CKD. Currently, the number of diabetes patients in China has exceeded 100 million (Li et al., 2020a), resulting in the increased incidence of DN, which has created a massive public health care burden. Several studies have focused on the roles of exosomal microRNAs in DN. In this section, we will discuss the growing body of research on microRNAs in DN.

The clinical manifestations and laboratory findings of DN are not specific. The diagnosis of DN currently relies primarily on a history of diabetes, proteinuria, and the progressive decline in renal function. Recently, researchers have found that changes in exosomal microRNA expression often occur early on in DN, even before the appearance of proteinuria and decreased renal function. Hence, exosomal microRNAs could be valuable for the early diagnosis of DN.

In a study of DN rat models, exosomal miR-451-5p was found to be significantly up-regulated in the urine 3–6 weeks after high glucose induction. This increase was accompanied by a 21% increase in mean proteinuria, although no change in tubulointerstitial fibrotic index (TFI) and glomerulosclerotic index (GI) was apparent. The TFI and GI remained unchanged until the 9th week, and the expression of miR-451-5p and miR-16 in renal tissues was negatively correlated with the TFI and GI at the 10th week (Mohan et al., 2016). This suggested that urinary exosomal miR-451-5p could be an early and sensitive non-invasive diagnostic indicator of DN and could also have some prognostic value. In another study on early DN, urinary exosomal miR-133b, miR-342, and miR-30a were shown to be significantly up-regulated in DN patients. The levels of these microRNAs were correlated with glycated hemoglobin, blood pressure, LDL, serum creatinine, the urinary albumin-to-creatinine ratio (ACR), and eGFR. Notably, 39.3, 19.6, and 17.9% of patients without proteinuria were positive for urinary exosomal miR-133b, miR-342, and miR-30a, respectively. Hence, these patients exhibited changes in microRNA levels before the onset of micro-albuminuria; thus, miR-133b, miR-342, and miR-30a could be used for the early detection of DN(54). In another clinical study, urinary exosomal miR-21-5p levels were found to be higher in patients with DN and CKD than in those with type 2 diabetes and normal renal function. In contrast, miR-30b-5p was down-regulated in DN and CKD patients. Both miR-21-5p and miR-30b-5p levels were significantly associated with serum creatinine levels. Hence, these two microRNAs appeared to be candidate biomarkers of renal function (Zang et al., 2019). Several other similar studies have also been conducted. In one study that included 23 patients with DN and a corresponding group of healthy volunteers, seven serum exosomal microRNAs (miR-1246, miR-642a-3p, miR-let-7c-5p, miR-1255b-5p, miR-let-7i-3p, miR-5010-5p, and miR-150-3p) were found to be up-regulated in DN patients. These microRNAs were significantly correlated with the level of proteinuria but not with eGFR, suggesting that they may be involved in the development of proteinuria in DN patients. Hence, these microRNAs may be candidate biomarkers for the diagnosis of DN as well as targets for therapy (Kim et al., 2019). In a larger study with more participants, urinary exosomal miR-15b, miR-34a, and miR-636 were found to be significantly up-regulated in patients with DN and positively correlated with serum creatinine levels and the ACR. Notably, the sensitivity of these microRNAs in diagnosing DN reached 100%, indicating that they may be key pathogenic factors and could serve as diagnostic markers of DN (Eissa et al., 2016b). However, the aforementioned studies could not demonstrate whether the microRNAs were associated with CKD or were specific for DN.

Lee et al. (Lee et al., 2020) examined patients with a clear biopsy-based diagnosis of DN and identified the differential expression of 72 urinary exosomal microRNAs. Of these, miR-188-5p showed the greatest up-regulation (Xue et al., 2018). The results suggested that besides being diagnostic markers of DN, these microRNAs could also be directly involved in the development and progression of DN. In addition, other studies also revealed the significant up-regulation of urinary exosomal miR-let-7c-5p (Li et al., 2018a), miR-362-3p, miR-877-3p, and miR-150-5p (Xie et al., 2017a) and the down-regulation of miR-15a-5p (Xie et al., 2017a), miR29c-5p, and miR-15b-5p (Li et al., 2018a) in patients with DN diagnosed by renal biopsy. Hence, these microRNAs may represent novel biomarkers for the early diagnosis of DN and help in monitoring the development of DN.

Some exosomal microRNAs have also been implicated in the development and progression of DN. The infiltration of inflammatory cells is a major pathological feature of various kidney diseases, including DN (Cao et al., 2015; Huen and Cantley, 2017). Macrophages differentiate into two types of cells under different pathophysiological conditions—M1 and M2 macrophages. M1 macrophages promote the development of kidney disease by secreting pro-inflammatory cytokines, while M2 macrophages seem to play an anti-inflammatory role (Shapouri-Moghaddam et al., 2018).

Podocyte injury is an early pathological feature of DN. In a study on the involvement of M2 macrophages in podocyte injury, exosomal miR-25-3p was delivered from M2 macrophages to podocytes. miR-25-3p attenuated high glucose-induced podocyte injury by directly binding to dual specificity phosphatase 1 (DUSP1), which promoted podocyte autophagy and prevented podocyte injury (Huang et al., 2020). This study provided a new option for the treatment of DN. Another study on the inflammatory response of renal proximal tubular epithelial cells (PTECs) provided a novel strategy for DN treatment. In this study, the inhibition of exosomal secretion from PTECs was found to promote the intracellular levels of miR-26a-5p. The intracellular miR-26a-5p bound to CHAC1, thereby inhibiting the CHAC1/NF-κB pathway (Li et al., 2020b), which is important for the inflammatory response (Liu et al., 2011). BMSCs were also found to play a key role in DN therapy (Sun et al., 2018). In one study, miR-let-7a was reported to be down-regulated in DN (Yan et al., 2016), but BMSC therapy could reverse this change by delivering miR-let-7a to renal tissues *via* exosomes. The overexpression of exosomal miR-let-7a was found to be negatively correlated with serum creatinine, BUN, TG, and TC levels. Further, it could inhibit the apoptosis of renal cells by targeting ubiquitin-specific protease 22 (USP22) (Mao et al., 2021), which regulates renal function in patients with DN (Huang et al., 2015). Podocytes typically show a series of morphological changes after injury, including hypertrophy, EMT, shedding, and apoptosis (Lal and Patrakka, 2018). Unfortunately, the pathogenesis of these processes remains to be fully understood. Adipose-derived stem cells (ADSCs) were shown to reverse EMT by delivering miR-215b to podocytes. This miR-215b could directly target zinc finger E-box-binding homeobox 2 (ZEB 2) (Jin et al., 2020).

DN is also characterized by increased interstitial fibrosis. An AAV with a miR23a/27a overexpressing construct was injected into the tibialis anterior muscle of mice. Thus, miR23a/27a-enriched exosomes were delivered to the kidneys *via* the circulation. miR-23a/27a overexpressed in the kidneys could attenuate renal fibrosis by reducing the expression of ECM proteins through Smad3 targeting (Zhang et al., 2018a). Similarly, ADSC-derived exosomes could attenuate DN by up-regulating miR-486 in renal cells, which could directly target Smad1 and then inhibit mTOR activation, increasing autophagy and reducing podocyte apoptosis (Jin et al., 2019).

## Exosomal microRNAs in acute kidney injury

AKI is a syndrome that occurs due to the rapid decline of renal function over a short period. It has multiple causes and is associated with significant morbidity and mortality (Negi et al., 2018).

### Exosomal microRNAs as biomarkers for the diagnosis and treatment of AKI

Exosomal microRNAs can also be used for the diagnosis and monitoring of AKI. Studies have shown that the etiology of AKI affects the levels of exosomal microRNAs. Yun et al. found that in patients with AKI due to scrub typhus, microRNA-21 levels were significantly increased in urinary exosomes. They were positively correlated with the total leukocyte count and negatively correlated with eGFR. These results suggested that miR-21 could serve as a diagnostic biomarker for AKI due to scrub typhus (Yun et al., 2021). In mice with hypoxia-induced acute tubular injury, miR-20a-5p levels were found to be significantly elevated in exosomes secreted from RTECs. Further, miR-20a-5p was shown to improve renal function by reducing serum creatinine and urea nitrogen levels, promoting endothelial cell proliferation, and protecting renal cells from apoptosis (Yu et al., 2020). In addition to aiding with the identification of AKI etiology, changes in exosomal microRNA expression could also help in determining the degree of renal injury and recovery in patients with AKI. During the early stages of injury, the urinary levels of exosomal miR-16-5p, miR-24-3p, and miR-200c-3p are significantly elevated. In contrast, urinary exosomal miR-9a, miR-141, miR-200a, miR-200c, and miR-429 are up-regulated at the early recovery stage. miRTarBase showed that these up-regulated microRNAs shared common mRNA targets, i.e., ZEB1 and ZEB2, which are well-known regulators of TGF-β1 signaling and are associated with renal fibrosis. Hence, the differential expression of these exosomal microRNAs suggested that they may be involved in the development of AKI and may also serve as biomarkers for the progression of kidney injury in AKI (Sonoda et al., 2019).

### Mechanistic role of exosomal microRNAs in the occurrence and development of AKI

**FIGURE 2:**
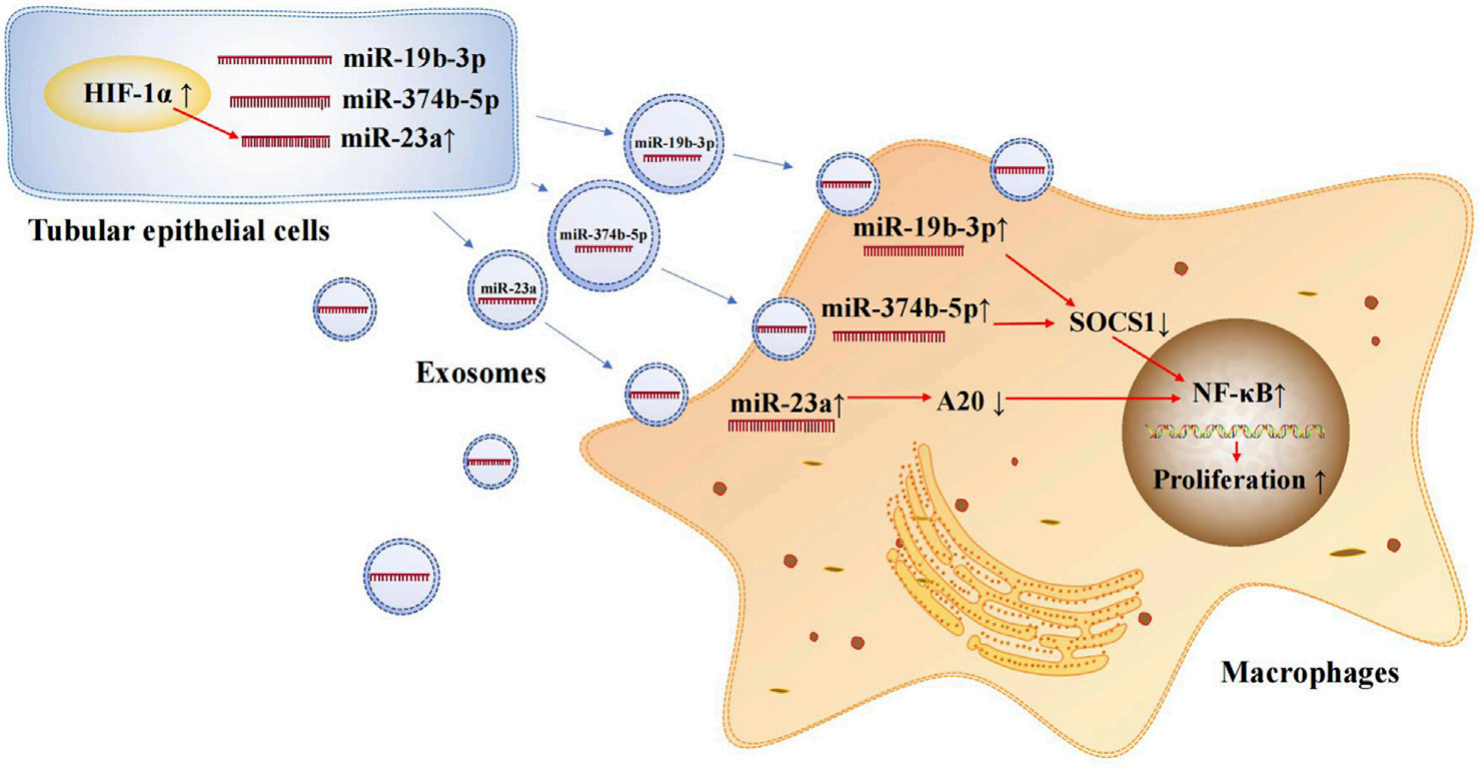
Exosomal microRNAs secreted by renal tubular epithelial cells activate M1 macrophages. Exosomal miR-374b-5p, miR-19b-3p and miR-23a secreted from RTECs promote the conversion of macrophages to M1 phenotype.

Exosomal microRNAs are involved in the occurrence and development of AKI. Tubulointerstitial inflammation is a common feature of AKI. Thus, it is essential to explore the mechanisms of tubulointerstitial inflammation for the treatment of AKI. Ischemia-reperfusion injury (IRI) is a common cause of AKI and leads to damage in RTECs. The damaged RTECs promote the conversion of macrophages to the M1 phenotype and secrete many inflammatory mediators, such as MCP-1, TNF-α, and IL-1β, thus promoting the renal inflammatory response (Meldrum et al., 2003; Lv et al., 2018b). One study found that exosomal microRNAs secreted from RTECs play an important role in promoting the conversion of macrophages to the M1 phenotype. During renal IRI, miR-374b-5p was found to be up-regulated in kidney-derived exosomes and was shown to bind directly to SOCS1 (Ding et al., 2020). Previous studies have confirmed that SOCS1 down-regulation can promote M1 macrophage activation (Liang et al., 2017). In addition, exosomal miR-19b-3p (Lv et al., 2020) and miR-23a (Li et al., 2019) secreted by RTECs are involved in the activation of M1 macrophages in murine models of AKI. In an AKI mouse model, miR-19b-3p showed the most significant up-regulation among 176 differentially expressed microRNAs. Further, it promoted M1 macrophage activation *via* the SOCS1/NF-κB signaling pathway (Lv et al., 2020). Under hypoxic conditions, hypoxia inducible factor-1α (HIF-1α) in RTECs induces miR-23a expression; subsequently, miR-23a-enriched exosomes are taken up by macrophages. miR-23a directly binds to the ubiquitin editor A20, which can target and regulate NF-κB. Accordingly, miR-23a activates M1 macrophages and induces inflammation (Li et al., 2019). The crossover mechanisms of these exosomal microRNAs in renal injury and tubulointerstitial inflammation are summarized in Figure 2. This information provides new insights into the involvement of exosomal microRNAs in AKI and motivates the exploration of therapeutic targets for ischemic-hypoxic renal injury.

The main pathological process of AKI involves damage to RTECs. Therefore, several studies have explored how RTEC apoptosis can be inhibited in AKI (Linkermann et al., 2014). Stem cell therapy (e.g., human urine-derived stem cells and mesenchymal stem cells) is highly effective in reducing tissue damage and accelerating tissue repair in AKI. It has now been revealed that the therapeutic effects of stem cells mainly result from their paracrine action (Bi et al., 2007). Exosomal microRNAs secreted by stem cells have been found to inhibit apoptosis by targeting key apoptosis molecules. Human urine-derived stem cells (USCs) are highly homologous to urinary system cells. One study found that the exosomal miR-216a-5p secreted by USCs inhibited cell apoptosis (Zhang et al., 2020a) and promoted renal repair in AKI (Tian et al., 2017). This was mainly because USC-derived exosomal miR-216a-5p directly targeted phosphatase and tensin homolog (PTEN) and inhibited apoptosis *via* the PTEN/Akt pathway (Zhang et al., 2020a). In addition, exosomes secreted by USCs were also found to up-regulate miR-146a-5p in RTECs, which could target interleukin-1 receptor-associated kinase (IRAK1), inhibiting cell apoptosis *via* the IRAK1/NF-κB signaling pathway (Li et al., 2020c). Similarly, human umbilical cord blood endothelial colony-forming cells (ECFCs) can secrete miR-486-5p-enriched exosomes. This miR-486-5p was found to directly bind to PTEN and inhibit the apoptosis of RTECs *via* the PTEN/Akt pathway (Vinas et al., 2016).

Sepsis is a systemic inflammatory response that occurs secondary to infection, and it can lead to multi-organ dysfunction. AKI is one of the most common and serious complications of sepsis (Bellomo et al., 2017). Exosomal miR-146b derived from human umbilical cord MSCs (HucMSCs) was found inhibit RTEC apoptosis *via* the IRAK1/NF-κB signaling pathway and ameliorate sepsis-induced AKI(93). A study found that remote ischemic preconditioning (rIPC) caused by transient ischemia and reperfusion of the femoral artery can protect against sepsis-induced AKI, and this effect is mediated by exosomal microRNAs. Serum exosomal miR-21 can be up-regulated in a HIF-1α-dependent manner after limb rIPC. In the kidneys, up-regulated exosomal miR-21 enters RTECs and then targets the downstream PDCD4/NF-κB and PTEN/AKT pathways, exerting anti-inflammatory and anti-apoptotic effects both *in vivo* and *in vitro*, thereby reducing sepsis-induced renal injury (Pan et al., 2019).

Endoplasmic reticulum stress is also involved in IRI-induced apoptosis. BMSCs inhibit renal cell apoptosis by secreting exosomes enriched with miR-199a-5p, which can directly inhibit binding immunoglobulin protein (BIP) in the early phase of reperfusion and exert a protective effect against renal ischemia-reperfusion injury (Wang et al., 2019c). All the aforementioned exosomal microRNAs can help inhibit apoptosis and contribute to kidney protection by targeting the key molecules of apoptotic pathways. Hence, they can be targeted to develop potentially viable strategies for the clinical treatment of AKI.

## Exosomal microRNAs in renal cell carcinoma

Renal cell carcinoma (RCC) is one of the most common cancers of the excretory system. The prognosis of RCC varies greatly according to its pathological type. Exosomal microRNAs could act as alternatives to invasive tumor biopsies and help in differentiating between benign and malignant tumors, thus aiding treatment selection (Dabestani et al., 2016).

RCC originates from renal epithelial cells, and clear cell RCC (ccRCC) is the most common subtype (Zhang et al., 2015). In a study with 82 ccRCC patients and 80 healthy individuals, serum exosomal miR-210 and miR-1233 were found to be elevated in ccRCC patients, regardless of the TNM stage. Moreover, the levels of these microRNAs became significantly lower after surgery. ROC analysis showed that the sensitivities of exosomal miR-210 and miR-1233 as diagnostic biomarkers for ccRCC were 70 and 81% and the specificities were 62.2 and 76%, respectively (Zhang et al., 2018b). This study suggested that the levels of circulating exosomal miR-210 and miR-1233 could be potential biomarkers for the diagnosis and clinical monitoring of ccRCC. Serum exosomal miR-210 was also found to be up-regulated in ccRCC patients in another study (Wang et al., 2018), showing a diagnostic sensitivity and specificity of 82.5 and 80.0%, respectively. In another study that included 22 RCC patients and 16 healthy controls, patients with RCC showed up-regulated exosomal miR-149-3p and miR-424-3p and down-regulated miR-92a-1-5p in the plasma. The diagnostic sensitivities of miR-92a-1-5p, miR-424-3p, and miR-149-3p were 87.5, 75.0, and 75.0%, and the specificities were 77.3, 81.8, and 72.7%, respectively (Xiao et al., 2020). Urinary exosomal miR-30c-5p also appeared to be a potential diagnostic biomarker for early ccRCC, and miR-30c-5p overexpression was shown to inhibit ccRCC progression *in vitro* and *in vivo* by targeting heat-shock protein 5 (HSPA5) (Song et al., 2019).

Xp11.2 translocation renal cell carcinoma (Xp11 tRCC) was once thought to be a rare form of RCC; however, it is now believed to account for 42% of all RCC cases in children and young adults (Cajaiba et al., 2018). Xp11 tRCC is characterized by a chromosomal translocation with a breakpoint in the *TFE3* gene on chromosome Xp11.2 (Kauffman et al., 2014). The early identification of these tumors is challenging. As a result, several patients show metastasis and progression at the time of diagnosis. In mouse models of Xp11 tRCC, urinary exosomal miR-204-5p is significantly up-regulated and is correlated with disease progression. Hence, this microRNA could be used as a biomarker for the early detection of Xp11 tRCC (Kurahashi et al., 2019).

Exosomal microRNAs can also be used to monitor therapeutic efficacy in patients with RCC. The cryoablation of renal cancer is a minimally invasive procedure (Kunkle and Uzzo, 2008; Ge et al., 2016). In one study, serum exosomal miR-126-3p, miR-17-5p, and miR-21-3p were found to rapidly decrease 1 day after treatment in the RCC resection group, suggesting that these microRNAs can directly reflect the effect of surgical tumor resection. Meanwhile, these three exosomal microRNAs also decreased significantly in the cryoablation and partial cryoablation groups for up to 7 days, and were significantly lower in the cryoablation group than in the partial cryoablation group. These results suggested that serum exosomal miR-126-3p, miR-17-5p, and miR-21-3p levels are correlated with the number of surviving tumor cells and may be sensitive candidates for assessing the effect of cryoablation (Zhang et al., 2018c). Changes in the levels of these circulating exosomal microRNAs could be used to monitor the regression of RCC. Exosomal miR-9-5p derived from renal cancer cells was found to promote the proliferation and invasion of tumor cells through SOCS4, which has a strong effect on the Janus kinase/signal transduction and activator of transcription (JAK/STAT) pathway (Song et al., 2020).

Despite the progress in the diagnosis and treatment of RCC, recurrence and distant metastases often occur and affect patient survival (Capitanio and Montorsi, 2016). Exosomal microRNAs could be used as potential prognostic biomarkers for RCC. In a study including patients with metastatic renal cancer, serum exosomal miR-26a-1-3p, miR-let-7i-5p, and miR-615-3p levels were found to be associated with overall survival. Furthermore, the prognostic value of miR-let-7i-5p was found to be better than that of the Memorial Sloan-Kettering Cancer Center (MSKCC) prognostic score. This suggested that exosomal miR-let-7i-5p has a potential value as a prognostic biomarker and could predict overall survival in patients with RCC (Du et al., 2017).

## Exosomal microRNAs in other kidney diseases

In addition to the previously mentioned disorders, exosomal microRNAs have also been shown to be involved in other kidney diseases. Since only few studies have examined these kidney diseases, we have discussed them together in this section.

### Other glomerular diseases

IgA nephropathy (IgAN) is the most common type of primary glomerulonephritis (Wyatt and Julian, 2013; Trimarchi et al., 2019). In a pilot study, urinary exosomal miR-204 was found to be significantly down-regulated in patients with IgAN. Although no difference in miR-204 expression was detected between IgAN and non-IgAN CKD controls, urinary miR-204 expression was lower in patients with IgAN at high risk of future progression than in those with a low risk of progression. These results suggested that urinary exosomal miR-204 could predict the risk of IgAN progression (Pawluczyk et al., 2021).

Lupus nephritis (LN), characterized by autoimmune glomerulonephritis, is one of the most common and serious complications of systemic lupus erythematosus and is associated with considerable morbidity and mortality (Borchers et al., 2012). Changes in exosomal microRNA expression were also found play a role in LN. Urinary exosomes of LN patients who responded after 12 months of treatment showed up-regulated levels of miR-31-5p, miR-107, and miR-135b-5p. These microRNAs were mainly produced in renal tubular cells and phagocytosed by endothelial and thylakoid cells. Then, they suppressed inflammation and the proliferation of thylakoid cells *via* HIF-1α inhibition (Garcia-Vives et al., 2020). Urinary exosomal miR-let-7a and miR-21 were differentially expressed in the active and remission phases of LN, revealing their value for monitoring LN activity (Tangtanatakul et al., 2019). The level of urinary exosomal miR-29c was found to be negatively correlated with indicators of glomerulosclerosis, indicating its potential as a new noninvasive marker of fibrosis progression in LN patients (Sole et al., 2015).

LN also involved podocyte injury (Rezende et al., 2014). Urinary exosomal miR-26a was found to be significantly up-regulated in patients with LN and was positively correlated with the level of proteinuria. In contrast, urinary exosomal miR-26a was significantly down-regulated in glomeruli and was observed to be associated with podocyte injury in mice with LN. Hence, miR-26a could be used as a marker for the diagnosis of LN as well as for monitoring podocyte injury (Ichii et al., 2014). Type IV lupus nephritis (LNIV) is a serious disease characterized by diffuse proliferative lesions. A cellular crescent (CC) found on renal biopsy is closely associated with rapid renal failure and is indicative of a poor prognosis (Cai et al., 2018; Zhang et al., 2018d). One study found that the levels of miR-3135b, miR-654-5p, and miR-146a-5p in urinary exosomes have potential value as novel non-invasive diagnostic markers for LNIV-CC (Li et al., 2018b).

Pediatric idiopathic nephrotic syndrome (INS) is a chronic glomerular disease commonly seen in children (Eddy and Symons, 2003). Urinary exosomal miR-194-5p, miR-146b-5p, miR-378a-3p, miR-23b-3p, and miR-30a-5p were found to be significantly up-regulated in children with INS and significantly down-regulated during clinical remission. Among these microRNAs, miR-194-5p and miR-23b-3p were significantly and positively correlated with 24-h urine protein concentrations. These urinary exosomal microRNAs could help in the diagnosis and monitoring of INS in children (Chen et al., 2019). Further, the changes in the levels of exosomal microRNAs differ depending on the pathological type of the disease. One study on patients with minimal change disease (MCD) and focal segmental glomerulosclerosis (FSGS) showed that the levels of miR-30b, miR-30c, miR-34b, miR-34c, and miR-342 in plasma exosomes and those of miR-1225-5p in urinary exosomes were higher in MCD patients than in FSGS patients and controls. In contrast, urinary levels of exosomal miR-1915 and miR-663 were lower and those of miR-155 was higher in FSGS patients than in MCD and controls (Ramezani et al., 2015). These studies suggest that exosomal microRNAs are an important adjunct for the pathological staging of INS and have important implications for the diagnosis and management of patients with contraindications to renal puncture.

### Kidney stones

There are several types of kidney stones. The most common component of kidney stones is calcium oxalate, and its deposition is a key factor for kidney stone formation in children (Kusmartsev et al., 2016; Zeng et al., 2017). Shi et al. reported that ADSC-derived exosomes enriched with miR-20b-3p could protect hyperoxaluric rats. Subsequently, their *in vitro* assays confirmed that miR-20b-3p could protect against kidney stones by inhibiting ATG7-mediated autophagy and TLR4-mediated inflammation (Shi et al., 2019).

### Hereditary kidney disease

Autosomal dominant polycystic kidney disease (ADPKD) is the most common inherited kidney disease (Ong et al., 2015). miR-194-5p was found to be down-regulated in urinary exosomes from patients with early ADPKD. Subsequent experiments showed that the decrease in miR-194-5p was associated with a significant reduction in the proliferation of human ADPKD cells *via* binding to PIK3R1 and the calcium-activated chloride channel anoctamin-1 (ANO1), resulting in cyst enlargement. Hence, miR-194-5p could serve as a target for preventing ADPKD progression and treating this condition (Magayr et al., 2020).

### Congenital kidney disease

Congenital hydronephrosis can cause acute or chronic damage to fetal kidneys, leading to neonatal death after birth. The prevention and timely treatment of congenital obstructive nephropathy is very challenging due to the lack of appropriate biomarkers. In one study, miR-942, miR-4289, miRPlus-A1073, and miR-195-3p were found to be up-regulated in exosomes derived from the amniotic fluid of fetuses with congenital hydronephrosis. The levels of another 35 exosomal microRNAs were found to be reduced. KEGG pathway analysis revealed that this down-regulation of exosomal miR-300 and miR-299-5p affected the Wnt/β-catenin pathway, creating significant implications for both the diagnosis and treatment of congenital hydronephrosis (Xie et al., 2017b).

### Kidney transplantation

Currently, kidney transplantation is a routine treatment for end-stage renal disease (Leeson and Desai, 2015). However, post-transplant renal insufficiency affects the survival rates of patients undergoing transplantation. Interstitial fibrosis and tubular atrophy (IF/TA) are the main causes of chronic graft dysfunction after kidney transplantation. As in other kidney diseases, exosomal microRNAs have also shown associations with kidney function after transplantation (Scian et al., 2011). One study showed that the plasma levels of exosomal miR-21 were elevated after kidney transplantation and were associated with high-grade IF/TA. This suggested that exosomal miR-21 levels in the plasma may be a better indicator of IF/TA than renal biopsy findings, facilitating earlier treatment (Saejong et al., 2022).

Other plasma-derived exosomal microRNAs could also be biomarkers for post-transplant renal function. Exosomal miR-21, miR-210, and miR-4639 levels in the plasma were found to be negatively correlated with eGFR and could help differentiate chronic graft dysfunction (eGFR <60 ml/min/1.73 m^2^) from normal graft function (eGFR >90 ml/min/1.73 m^2^) (Chen et al., 2020). Tacrolimus modulates the suppression of regulatory T cells during the allogenic immune response after renal transplantation (Wong et al., 2017); exosomal microRNAs can enable the assessment of tacrolimus efficacy as well as post-transplant renal function. In a study conducted among tacrolimus-treated renal transplant patients, 16 urinary exosomal microRNAs were found to be differentially expressed after tacrolimus-based therapy. Among these microRNAs, miR-155-5p was significantly up-regulated, while miR-223-3p and miR-1228-3p were significantly down-regulated. Urinary exosomal miR-155-5p and miR-2233 were correlated with the dose of tacrolimus, miR-223-3p was correlated with serum creatinine levels, and miR-223-3p and miR-1228-3p were correlated with leukocyte counts (Freitas et al., 2020).

Delayed graft function (DGF), a manifestation of acute renal failure, occurs in approximately 2–50% of kidney transplant patients in the first week after surgery and affects survival rates (Yarlagadda et al., 2009). In one study, exosomal microRNAs (miR-33a-5p_R-1, miR-98-5p, and miR-151a-5p) were significantly up-regulated in the plasma obtained from kidney recipients with DGF. Among them, miR-151a-5p was positively correlated with serum creatinine, urea nitrogen, and uric acid levels in post-transplant kidney recipients during the first week after kidney transplantation. These microRNAs were involved in the development of DGF and have value as diagnostic biomarkers and therapeutic targets (Wang et al., 2019d).

## Discussion

This review summarized recent findings regarding the roles of exosomal microRNAs in various kidney diseases. Kidney diseases are accompanied by changes in the levels of exosomal microRNAs, and these levels also differ depending on the cause and stage of the disease. Table 1 lists several exosomal microRNAs that are altered in kidney diseases. This information could provide new avenues for the diagnosis of kidney diseases. Interestingly, miR-26a, miR-150-3p, miR-let-7c-5p, and miR-194-5p were examined in multiple studies. Urinary exosomal miR-26a was found to be down-regulated in two studies (Ichii et al., 2017; Ichii et al., 2018) examining cats and dogs with CKD. However, it was found to be up-regulated in patients with LN(119). Whether this difference is due to the species or the primary disease itself requires further research. miR-150-3p and miR-let-7c-5p were found to be up-regulated in both serum-derived and urinary exosomes in patients with DN(56, 58, 60), showing a greater diagnostic value and a better correlation with the development of DN. miR-194-5p was found to be down-regulated in serum exosomes in CKD(30), up-regulated in urinary exosomes in pediatric INS(124), and down-regulated in urinary exosomes in ADPKD(130). Owing to these differences in expression across different kidney diseases, exosomal miR-194-5p could be valuable in differential diagnosis. Its mechanisms of action are expected to be intriguing and will require further exploration.

**TABLE 1:** Changes in the levels of exosomal microRNAs in different renal diseases.

Diseases	Derived from	ncRNAs	Changes
CKD	Urine	miR-451(16)	up-regulated
		miR-181a Khurana et al., (2017)	down-regulated
		miR-let-7b, miR-22, miR-26a^a^, Ichii et al., (2018)	
		miR-26a^a^, miR-10a/b Ichii et al., (2017)	
	Serum	miR-192-5p, miR-194-5p^a^, miR-802-5p, miR-143-3p Liu et al., (2020)	down-regulated
DN	Urine	miR-451-5p, miR-16(53)	up-regulated
		miR-133b, miR-342, miR-30a Eissa et al., (2016a)	
		miR-21-5p Zang et al., (2019)	
		miR-15b, miR-34a, miR-636(57)	
		miR-188-5p, miR-150-3p^a^, miR-760, miR-3677-3p, miR-548ah-3p, miR-548p, miR-320e, miR-23c Lee et al., (2020)	
		miR-let-7c-5p^a^ Li et al., (2018a)	
		miR-362-3p, miR-877-3p, miR-150-5p Xie et al., (2017a)	
		miR-30b-5p Zang et al., (2019)	down-regulated
		miR-133a-3p, miR-153-3p Lee et al., (2020)	
		miR-15a-5p Xie et al., (2017a)	
		miR-29c-5p, miR-15b-5p Li et al., (2018a)	
	Serum	miR-1246, miR-642a-3p, miR-let-7c-5p^a^, miR-1255b-5p, miR-let-7i-3p, miR-5010-5p, miR-150-3p^a^, miR-4449(56)	up-regulated
AKI	Urine	miR-21(77)	up-regulated
		miR-16-5p, miR-24-3p, miR200c-3p (early injury state), miR-9a, miR-141, miR-200a, miR-200c, miR-429(recovery state) Sonoda et al., (2019)	
ccRCC	Serum	miR-210, miR-1233	up-regulated
	Plasma	miR-149-3p, miR-424-3p Xiao et al., (2020)	up-regulated
		miR-92a-1-5p Xiao et al., (2020)	down-regulated
Xp11 tRCC	Urine	miR-204-5p Kurahashi et al., (2019)	up-regulated
IgAN	Urine	miR-2045p Kurahashi et al., (2019)	down-regulated
LN	Urine	miR-26a^a^ Ichii et al., (2014)	up-regulated
Pediatric INS	Urine	miR-194-5p^a^, miR-146b-5p, miR-378a-3p, miR-23b-3p, miR-30a-5p Chen et al., (2019)	up-regulated
Congenital hydronephrosis	Amniotic fluid	miR-942, miR-4289, miRPlus-A1073, miR-195-3p Xie et al., (2017b)	up-regulated
ADPKD	Urine	miR-194-5p^a^ Magayr et al., (2020)	down-regulated

a Same exosomal microRNAs, are detected differentially expressed in multiple researches.

As discussed in this review, exosomal microRNAs have also been implicated in the development of kidney diseases. They are known to participate in processes such as apoptosis, proliferation, autophagy, inflammatory responses, and EMT. We summarized some of the currently known roles of exosomal microRNAs in kidney diseases in Table 2. These findings shed light on the pathogenesis of these diseases and provide directions for the development of molecular therapies.

**TABLE 2:** Mechanistic roles of exosomal microRNAs in different kidney diseases.

Diseases	ncRNAs	Derived from	Targets or signal pathways	Pathological processes	Functions	Species	References
CKD	miR-let-7c	MSCs	TGF-βR1/type IV α1 collagen, *α*-SMA	Renal fibrosis	Fibrosis inhibition	Rat	Wang et al. (2016)
CKD	miR-29a	Serum/intramuscular injection	YY1, TGF-β3	Renal fibrosis	Fibrosis inhibition	Mouse	Wang et al. (2019a)
CKD	miR-26	Serum	CTGF, TGF-β1	Renal fibrosis	Fibrosis inhibition	Mouse	Zhang et al. (2019b)
CKD	miR-26a-5p^a^	Intramuscular injection	IGF-1/Akt/FoxO	Renal fibrosis	Fibrosis inhibition	Mouse	Wang et al. (2019b)
CKD	miR-150-5p	Tubular cells	SOCS1	Renal fibrosis	Promotes fibroblast activation and accelerate the development of renal fibrosis	Mouse, rat	Zhou et al. (2021)
CKD	miR-381-3p	BMSCs	NFAT5	Apoptosis	Inhibits apoptosis and alleviates vascular calcification	Rat, human	Liu et al. (2022)
CKD	miR-21^a^	Tubular cells	PTEN/Akt	Renal fibrosis	accelerate the	Mouse, rat	Zhao et al. (2021)
					development of renal fibrosis		
CKD	miR-186-5p	MSCs	Smad 5	Renal fibrosis	Fibrosis inhibition	Mouse, rat	Yang et al. (2022)
DN	miR-23a/27a	intramuscular injection	Smad 2/3	Renal fibrosis	Fibrosis inhibition	Mouse	Zhang et al. (2018a)
DN	miR-26a-5p^a^	PTECs	CHAC1/NF-κB	Inflammation	Inhibits inflammation	Mouse, human	Li et al. (2020b)
DN	miR-let-7a	BMSCs	USP22	Inflammation	Inhibits apoptosis	Rat	Mao et al. (2021)
DN	miR-215-5p	ADSCs	ZEB2	EMT	Inhibits EMT	Mouse	Jin et al. (2020)
DN	miR-486	ADSCs	Smad 1/mTOR	Autophagy	Promotes autophagy	Mouse	Jin et al. (2019)
DN	miR-25-3p	M2 macrophages	DUSP1	Autophagy	Promotes autophagy	Mouse	Huang et al. (2020)
AKI	miR-374b-5p	TECs	SOCS1	Inflammation	Promotes M1 macrophage activation	Mouse	Ding et al. (2020)
AKI	miR-146a-5p	USCs	IRAK1/NF-κB	Inflammation	Inhibits inflammation	Rat	Li et al. (2020c)
AKI due to sepsis	miR-146b	HucMSCs	IRAK1/NF-κB	Inflammation	Inhibits inflammation	Mouse	Zhang et al. (2020b)
AKI due to sepsis	miR-21^a^	Serum	HIF-1α/miR21/PDCD4/NF-κB	Inflammation	Inhibits inflammation	Mouse	Pan et al. (2019)
AKI due to sepsis	miR-21^a^	Serum	HIF-1α/miR21/PTEN/Akt	Apoptosis	Inhibits apoptosis	Mouse	Pan et al. (2019)
AKI	miR-216-5p	USCs	PTEN↓/Akt↑/Caspase3↓	Apoptosis	Inhibits apoptosis	Human,rat	Zhang et al. (2020a)
AKI	miR-199-5p	BMSCs	BIP	Apoptosis	Inhibits endoplasmic reticulum stress and suppresses apoptosis	Mouse	Wang et al. (2019c)
AKI	miR-486-5p	ECFCs	PTEN/Akt	Apoptosis	Inhibits apoptosis	Mouse	Vinas et al. (2016)
Renal tubular interstitial inflammation	miR-19b-3p^a^	TECs	SOCS1/NF-κB	Inflammation	Promotes M1 macrophage activation	Mouse	Lv et al. (2020)
Renal tubular interstitial inflammation	miR-23a	TECs	HIF-1α/miR23a/A20/NF-κB	Inflammation	Promotes M1 macrophage activation	Mouse	Li et al. (2019)
RCC	miR-19b-3p^a^	CSCs	PTEN	EMT	Promotes EMT and migration of cancer cells	Human	Wang et al. (2019e)
RCC	miR-30c-5p	Urine	HSPA5	Proliferation	reduces cell viability and colony formation efficiency	Human	Song et al. (2019)
RCC	miR-9-5p	Serum	SOCS4/JAK-STAT	Proliferation	Promotes cells proliferation and migration	Human	Song et al. (2020)
Kidney stones	miR-20b-3p^a^	ADSCs	TLR4/NF-κB	Inflammation	Inhibits inflammation	Rat	Shi et al. (2019)
Kidney stones	miR-20b-3p^a^	ADSCs	ATG7, TLR4/NF-κB	Autophagy	Inhibits autophagy	Rat	Shi et al. (2019)
LN	miR135-b/miR-107/miR-31	Tubular cells	HIF-1α/(CXCL1, CCL3, CCL2), IL6, VCAM-1	Inflammation	Inhibits inflammation	Human	Garcia-Vives et al. (2020)
ADPKD	miR-194-5p	Urine	PIK3R1, anoctamin-1 (ANO1)	Proliferation	Inhibits the expansion of the cyst	Human	Magayr et al. (2020)

a Same exosomal microRNAs, are involved in different pathological processes of kidney diseases.

Some exosomal microRNAs are involved in multiple diseases and act through various signaling pathways. For example, miR-26a-5p can target IGF-1 during the pathogenesis of renal fibrosis (Wang et al., 2019b) and target CHAC1 while mediating the inflammatory response in DN(66). In addition, miR-21, which targets PTEN, is involved in renal fibrosis in CKD(33) and also targets PDCD4 to mediate the inflammatory response in AKI(92). Different kidney diseases may be associated with the same pathological changes, and microRNAs show some degree of synergistic or antagonistic effects in these processes. For example, exosomal miR-23a/27a (Zhang et al., 2018a) and miR-let-7c (Wang et al., 2016), miR-29(42), miR-26(48), and miR-186-5p (Yang et al., 2022) play an anti-fibrotic role in patients with DN and CKD, respectively. In contrast, miR-150-5p (Zhou et al., 2021) and miR-21(33) promote fibrosis in patients with CKD. Similarly, miR-374b-5p (Ding et al., 2020), miR-19b-3p (Lv et al., 2020), and miR-23a (Li et al., 2019) promote the activation of M1 macrophages and exacerbate the inflammatory response, while miR-26a-5p and miR-20b-3p exert anti-inflammatory effects in patients with DN(66) and kidney stones (Shi et al., 2019), respectively.

Together, these findings demonstrate that the roles of exosomal microRNAs are complex and need further elucidation. Additionally, many questions remain to be addressed. It is currently unclear why the levels of microRNAs differ between bodily fluids such as urine and blood in patients with the same disease (Fan et al., 2019; Perez-Hernandez et al., 2021). Furthermore, in some cases, the changes in microRNA levels are not consistent even in the same body fluid. For example, studies have shown that some microRNAs could be detected in the plasma but not in plasma-derived exosomes (Saejong et al., 2022). The mechanisms underlying these differences still need further research. The transport of exosomal microRNAs also varies. The current evidence suggests that exosomal microRNAs are transported across different organs through the circulation (Gao et al., 2020). However, there may also be paracrine interactions between neighboring tissues (Liang et al., 2019). The in-depth examination of these questions could help achieve targeted microRNA transport to specific organs and develop molecular therapies for kidney diseases.
